# Different Apoptotic Pathways Activated by Oxaliplatin in Primary Astrocytes *vs.* Colo-Rectal Cancer Cells

**DOI:** 10.3390/ijms16035386

**Published:** 2015-03-09

**Authors:** Matteo Zanardelli, Laura Micheli, Raffaella Nicolai, Paola Failli, Carla Ghelardini, Lorenzo Di Cesare Mannelli

**Affiliations:** 1 Department of Neuroscience, Psychology, Drug Research and Child Health – Neurofarba – Pharmacology and Toxicology Section, University of Florence, Florence 50139, Italy; E-Mails: matteo.zanardelli@unifi.it (M.Z.); laura.micheli@unifi.it (L.M.); paola.failli@unifi.it (P.F.); carla.ghelardini@unifi.it (C.G.); 2 Sigma-Tau Industrie Farmaceutiche Riunite S.p.A., Via Pontina km 30,400, I-00040 Pomezia, Rome 00040, Italy; E-Mail: Raffaella.Nicolai@sigma-tau.it

**Keywords:** glia, HT-29, neuropathic pain, intrinsic apoptotic pathway, extrinsic apoptotic pathway, oxaliplatin

## Abstract

Oxaliplatin-based chemotherapy improves the outcomes of metastatic colorectal cancer patients. Its most significant and dose-limiting side effect is the development of a neuropathic syndrome. The mechanism of the neurotoxicity is unclear. The limited knowledge about differences existing between neurotoxic and antitumor effects hinders the discovery of effective and safe adjuvant therapies. *In vitro*, we suggested cell-specific activation apoptotic pathways in normal nervous cells (astrocytes) *vs.* colon-cancer cells (HT-29). In the present research we compared the apoptotic signals evoked by oxaliplatin in astrocytes and HT-29 analyzing the intrinsic and extrinsic apoptotic pathways. In astrocytes, oxaliplatin induced a mitochondrial derangement measured as cytosolic release of cytochrome C, increase in superoxide anion levels and decreased expression of the antiapoptotic protein Bcl-2. Caspase-8, a main initiator of the extrinsic process remained unaltered. On the contrary, in HT-29 oxaliplatin increased caspase-8 activity and Bid expression, thus activating the extrinsic apoptosis, while the Bcl-2 increased expression blocked the mitochondrial damage. Data suggest the preferred activation of the intrinsic apoptosis as oxaliplatin damage signaling in normal nervous cells. The extrinsic pathway prevails in tumor cells indicating a possible strategy for planning new molecules to treat oxaliplatin-dependent neurotoxicity without negatively influence chemotherapy.

## 1. Introduction

Oxaliplatin was successfully introduced for the management of advanced colorectal cancer, the second leading cause of cancer death in Western countries [1,2]. This antineoplastic agent differs from previous platinum compounds for the configuration of the amino substituents [3,4] and, characteristically, its major dose-limiting side effect is neurotoxicity that leads to the development of peripheral neuropathy [5,6]. The poor knowledge about the mechanisms of oxaliplatin neurotoxicity limits the development of effective adjuvant therapies thus making chemotherapy-induced neuropathies an unmet medical need. Moreover, the pharmacological approach is complicated by the fundamental necessity to not interfere with the antitumoral effects.

Recently, we highlighted the redox unbalance as a target for the management of oxaliplatin neurotoxicity, and the natural antioxidant compound silibinin was suggested to prevent nervous damage and pain in a rat model of oxaliplatin neuropathy [7,8]. In a cellular model of oxaliplatin-neurotoxicity (primary astrocyte cell culture), silibinin, as well as α-tocopherol, exerted cytoprotective properties reducing the oxidative damage and limiting the activation of caspase-3, the downstream effector of apoptotic processes [9]. Interestingly, antioxidants were unable to reduce caspase-3 activation induced by oxaliplatin in the human adenocarcinoma colorectal cancer cell line HT-29 [9]. The different effect observed in astrocytes and in HT-29 lead us to hypothesize that oxaliplatin may evoke distinct apoptotic signals in normal *vs.* tumoral cells. On the other hand, the intrinsic and the extrinsic apoptotic pathways mediated by a mitochondrial derangement and by death receptors, respectively, have as common effector caspase 3 [10,11].

Aimed to individuate new and specific biological targets for the treatment of oxaliplatin neurotoxicity, specific markers of the two apoptotic pathways (extensively reviewed in [12]) were studied in primary cultured astrocytes in comparison with HT-29 cells. In particular, the mitochondrial dysfunction was studied by measuring the release of cytochrome C from mitochondria to the cytosol, the superoxide anion (O_2_^.−^) levels [13,14,15] and the expression of the antiapoptotic protein Bcl-2 [16]. Moreover, the protein expression levels were evaluated for the initiator of the extrinsic apoptotic process death receptor 5 (DR5) [17,18] and Bid, pro-apoptotic protein activated by caspase-8 and able to transfer the apoptotic information to the intrinsic process [19]. Finally, the activation of caspase-8, central hallmark of the extrinsic pathway was measured [11,20].

## 2. Results

Aimed to evaluate the regulation of the apoptotic processes mediated by oxaliplatin, specific effectors of the intrinsic and extrinsic apoptotic pathways were measured in primary rat astrocytes in comparison to HT-29 cells. Oxaliplatin concentration was chosen on the basis of previous published data [9]. Moreover, the comparison of astrocyte and HT-29 cell viability, after 24 h incubation with increasing concentrations of oxaliplatin, revealed a similar response in the different cell types (Supplementary Material, Table S1). The treatment with oxaliplatin 100 µM for 8 h did not alter cell viability, whereas is allows observing increased caspase-3 activity in astrocytes as well as in HT-29. The pro-apoptotic effect of oxaliplatin was comparable in both cell types [9].

**Figure 1:**
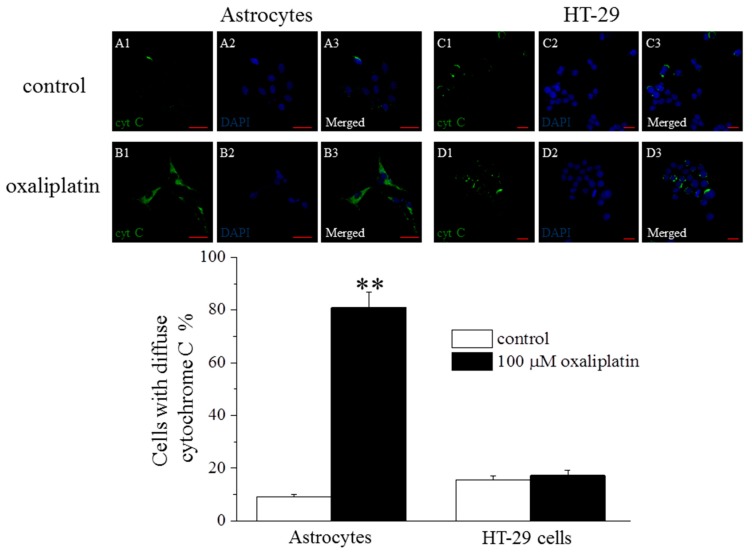
Cytosolic release of cytochrome C. Astrocytes (5 × 10^4^ cells/slide) and HT-29 (5 × 10^4^ cells/slide) were exposed to 100 μM oxaliplatin for 8 h. Specimens were stained with anti-cytochrome C and a secondary antibody conjugated with Alexa Fluor 488 (green) and DAPI (blue) for nucleus visualization. (**A1**–**A3**) control astrocytes; (**B1**–**B3**) oxaliplatin-treated astrocytes; (**C1**–**C3**) control HT-29; (**D1**–**D3**) oxaliplatin-treated HT-29. Calibration: 20 µm. Bars represent the mean ± S.E.M of cells displaying a diffuse cytosolic distribution of cytochrome C as percentage of total analyzed cells. Cells were counted using the “cell counter” plugin of ImageJ 1.33, free-share image analysis software (ImageJ, NIH, Bethesda, MD, USA). At least three fields (40X 0.75NA objective) per slide and two slides for each condition were analyzed, repeating the experiment three times. ** *p* < 0.01 *vs*. control.

In astrocytes, 8 h incubation with 100 µM oxaliplatin, affected mitochondrial functionality. The immunolabeling of cytochrome C displayed a punctuate staining in control condition that evolved in a diffuse cytosolic pattern after oxaliplatin treatment (Figure 1).

The release of cytochrome C from mitochondria to the cytosol was observed in 197 out of 247 treated cells and in 21 out of 253 control cells. On the contrary, oxaliplatin (100 µM, 8 h) did not alter cytochrome C localization in HT-29 (Figure 1). 

**Figure 2:**
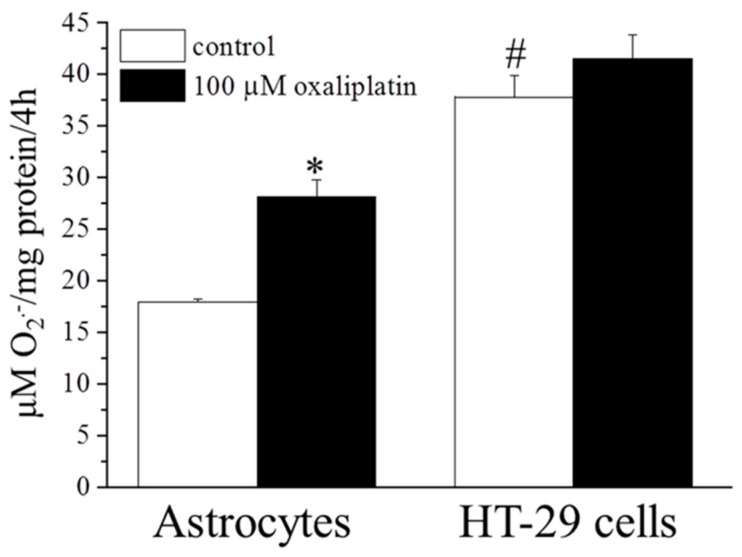
O_2_^.−^ concentrations. Astrocytes (5 × 10^5^ cells/well) and HT-29 (3 × 10^5^ cells/well) were exposed to 100 μM oxaliplatin for 4 h. O_2_^.−^ concentration was evaluated by cytochrome C assay. The nonspecific absorbance was measured in the presence of superoxide dismutase (SOD; 300 mU/mL) and subtracted from the total value. Values are expressed as µM/mg protein/4 h. Bars represent the mean ± S.E.M. of three experiments. * *p* < 0.05 *vs*. control and ^#^ *p* < 0.05 *vs.* astrocytes control.

In glial cells the mitochondrial alterations were also highlighted by measuring the redox unbalance. Superoxide anion production (O_2_^.−^) was increased by oxaliplatin (100 µM, 4 h) by about 1.5 times (in comparison to the basal level of control condition, 17.9 ± 0.3 µM/mg protein/4 h; Figure 2).

In HT-29 cells, the chemotherapic agent did not induce any increase in superoxide anion level as measured in astrocyte cultures. To note, the O_2_^.−^ basal level in the tumoral cells was significantly higher than those detected in the astrocyte cultures (37.8 ± 2.1 µM/mg protein/4 h; Figure 2).

**Figure 3:**
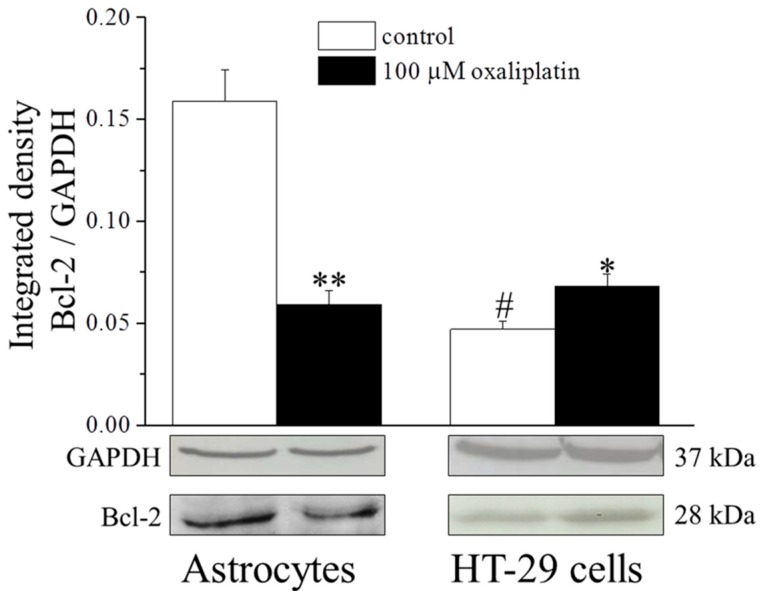
Bcl-2 protein expression. Astrocytes (10^6^ cells/flask) and HT-29 (8 × 10^5^ cells/flask) were incubated with 100 μM oxaliplatin for 8 h and then cell lysates were analyzed by Western blot. Densitometric analysis (**top**) and representative immunoblot (**bottom**) are shown. Glyceraldehyde-3-Phosphate Dehydrogenase (GAPDH) was used as loading control and GAPDH normalization was performed for each sample. Values are expressed as integrated density making the ratio between Bcl-2 and GAPDH specific band intensities. Bars represent the mean ± S.E.M. of three experiments. * *p* < 0.05 *vs*. control, ** *p* < 0.01 *vs*. control and ^#^ *p* < 0.05 *vs*. astrocytes control.

Evaluating protein expression by Western blot analysis, in basal conditions Bcl-2 was higher in astrocytes as compared to HT-29 (Figure 3). Incubation with oxaliplatin (100 µM, 8 h) reduced at about 63% Bcl-2 protein expression in astrocytes, whereas it increased Bcl-2 protein level up to 145.6% ± 12.1% in HT-29 cells (Figure 3).

**Figure 4:**
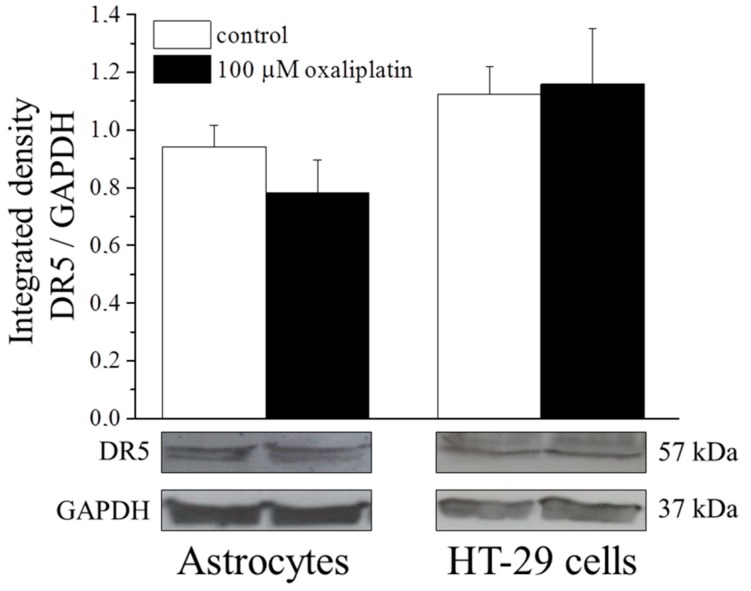
Death receptor 5 (DR5) protein expression. Astrocytes (10^6^ cells/flask) and HT-29 (8 × 10^5^ cells/flask) were incubated with 100 μM oxaliplatin for 8 h and then Western blot analysis were performed. Densitometric analysis (**top**) and representative immunoblot (**bottom**) are shown. GAPDH was used as loading control and GAPDH normalization was performed for each sample. Values are expressed as integrated density making the ratio between DR5 and GAPDH specific band intensities. Bars represent the mean ± S.E.M. of three experiments.

The analysis of extrinsic pathway parameters revealed a selective activation in HT-29 cell culture. The incubation (8 h) with 100 µM oxaliplatin did not significantly alter the protein expression of the DR5 receptor neither in astrocytes nor in HT-29 (Figure 4).

**Figure 5:**
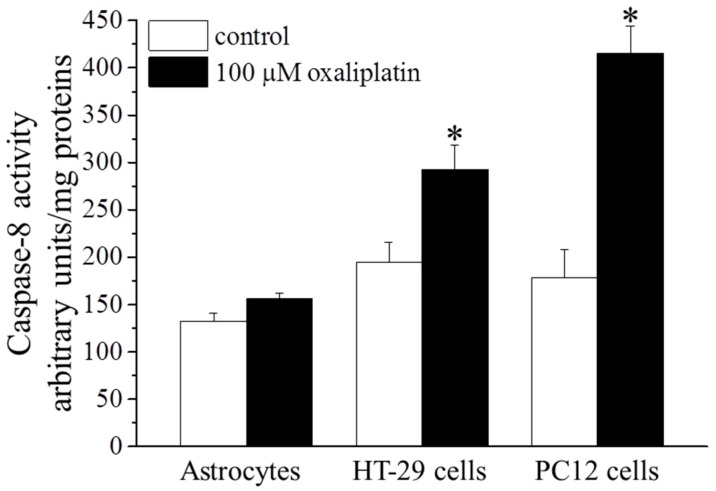
Caspase-8 activity. Astrocytes (5 × 10^5^ cells/well), HT-29 (3 × 10^5^ cells/well) and PC12 (3 × 10^5^ cells/well) were treated with 100 μM oxaliplatin for 8 h. Caspase-8 activity was expressed as fluorescent arbitrary unit/mg protein. Bars represent the mean ± S.E.M. of three experiments. * *p* < 0.05 *vs.* control.

We performed experiments aimed to measure caspase-8 activity. As shown in Figure 5, caspase-8 activity in astrocyte was 132.3 ± 8.9 arbitrary units/mg proteins in control condition and was not changed by oxaliplatin. On the contrary, in HT-29 oxaliplatin increased the enzyme activity up to 292.9 ± 25.9 arbitrary units/mg proteins from the control condition of 194.5 ± 21.4 arbitrary units/mg proteins.

Also oxaliplatin increased caspase-8 activity in the rat adrenal pheochromocytoma cell line PC12 from the control value of 178.6 ± 29.6 arbitrary units/mg proteins to 415.8 ± 28.7 arbitrary units/mg proteins (Figure 5).

**Figure 6:**
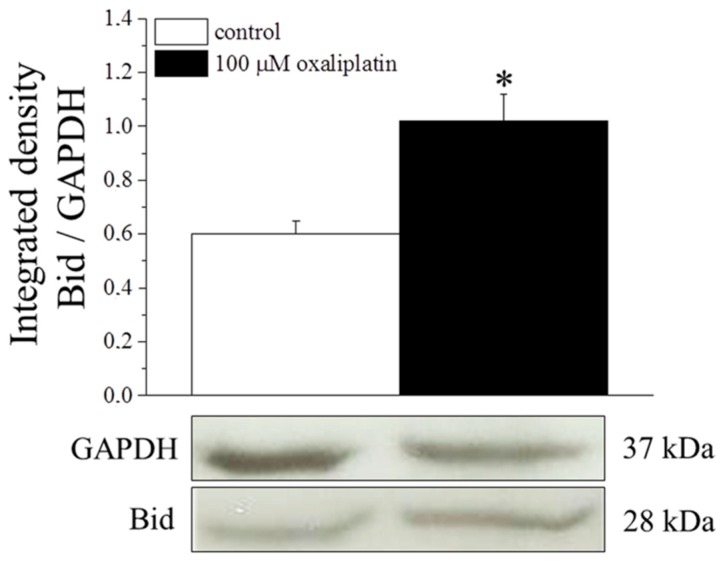
Bid protein expression. HT-29 (8 × 10^5^ cells/flask) were incubated with 100 μM oxaliplatin for 8 h and then cell lysates were analyzed by Western blot. Densitometric analysis (**top**) and representative immunoblot (**bottom**) are shown. GAPDH was used as loading control and GAPDH normalization was performed for each sample. Values are expressed as integrated density making the ratio between Bid and GAPDH specific band intensities. Bars represent the mean ± S.E.M. of three experiments. * *p* < 0.05 *vs.* control.

Figure 6 shows protein expression levels of Bid evaluated by Western blot. In HT-29, oxaliplatin (100 µM, 8 h) increased Bid by about 70%. In astrocyte cells, Bid was undetectable in control condition as well as after oxaliplatin treatment.

## 3. Discussion

Neuropathic syndrome development is one of the main adverse drug reactions that negatively influence the anticancer therapy and drastically reduce the quality of life of the oncologic patients [1,5]. The lack of knowledge about the pathological events at the base of the neurodegenerative phenomenon is primarily responsible for the limited therapeutic resources. The available symptomatic drugs are scarcely effective and the identification of active disease-modifying agents remains the goal of the research in this field. On the other hand, the most important feature of antineuropathic agents candidate to treat cancer patients, is the lack of interaction with chemotherapies maintaining a full effectiveness of the anti-neoplastic potential. A limited knowledge of the distinctions between the biological alterations promoted by the anticancer agents in normal nervous cells and in tumor cells strongly impairs the development of targeted adjuvant therapies.

In the present research, the attention has been focused on oxaliplatin, showing that the apoptosis-inducing mechanism is preferentially mediated in astrocytes by the intrinsic pathway and by the extrinsic in colon cancer cell HT-29.

The intrinsic pathway is promoted by a mitochondrial alteration characterized by membrane potential changes, release of cytochrome C and redox unbalance [21,22]. In normal glial cells, oxaliplatin promotes the release of cytochrome C from mitochondria to the cytosol increasing the number of cells showing a diffuse immunoreactivity. Accordingly, Zheng *et al.* [23] described an oxaliplatin-mediated increase of swollen and vacuolated mitochondria in peripheral nerve axons of neuropathic rats [24]. Mitochondria represent the major source of intracellular ROS, which are by-products of oxidative phosphorylation. ROS excess, or the limited counterbalance by endogenous antioxidant defenses, may damage lipids, proteins, DNA, and mitochondria itself [24]. Oxidative stress can activate the mitochondrial permeability transition pore (mtPTP) creating an open channel across the mitochondrial inner and outer membranes which permits the free diffusion of molecules with a molecular weight lesser than 1500 Da. The resulting collapse of *trans*-membrane electrochemical gradient and the swelling of mitochondria cause the release of cytochrome C [24,25]. Among other ROS, O_2_^.−^ may directly lead to reduced complex I activity, limiting drastically the metabolic role of the organelle [26,27,28]. In this view, the increased O_2_^.−^ levels registered in nervous cells treated with oxaliplatin [9] and confirmed in the present results, underlines the mitochondrial suffering.

The extent of the intrinsic pathway activation appears marked in astrocytes since levels of the antiapoptotic protein Bcl-2 is reduced after oxaliplatin treatment. Bcl-2 belongs to the Bcl-2 protein superfamily composed by anti-apoptotic and pro-apoptotic factors [26]. Bcl-2 is localized on the external mitochondrial membrane and prevents the release of pro-apoptotic molecules (such as cytochrome C) from the mitochondria to the cytosol [29,30] limiting the voltage dependent anion channel formation and the dysregulation of the mitochondrial membrane potential [31,32]. Normally, astrocytes express higher steady-state levels of Bcl-2 than neurons [33] suggesting a low vulnerability of astrocytes. Furthermore, in astrocytes oxaliplatin does not increase the expression of the pro-apoptotic protein Bid (able to activate the intrinsic apoptotic process by inducing the cytosolic release of cytochrome C) [34], and Bid basal levels are under the detection threshold. The low basal level of Bid in astrocytes agrees with Harrison *et al.* [33].

Under physiological conditions, glial cells exert neuroprotective effects by providing neurons with substrates for oxidation, regulating the levels of neurotoxic molecules like glutamate [35] and free radicals [36,37]. The induction of apoptosis, the dysregulation of superoxide anion homeostasis [9], and the reduction of Bcl-2 levels in astrocytes suggest that the neurotoxic effects of oxaliplatin derive, at least in part, from an impairment of glial neuroprotective activity.

Caspase-8 is one of the principal initiator of the extrinsic process and directly activates the effector caspase-3 [38]. The two major pathways to apoptosis are largely independent, although in certain cell types (e.g., hepatocytes) may intersect. Indeed, caspase-8 can process the pro-apoptotic Bid into its active truncated form transferring the apoptotic information to the mitochondrial process [39]. On the other hand, to prevent catastrophic unscheduled cell death, both pathways are tightly regulated, at multiple steps. Present data show that the apoptotic process activated by oxaliplatin in astrocytes is completely independent from this apoptotic mediator, whereas the anticancer agent significantly increases caspase-8 activity in the human colon cancer cell line HT-29 and rat PC12 cells (ruling out the possibility that a species-dependent diversity may influence the results). In HT-29 cells, the expression of Bid is increased after oxaliplatin treatment, confirming the activation of the extrinsic pathway. Moreover, Bid seems unable to cross-activate the intrinsic process since cytochrome C is not significantly released in the cytosol in HT-29. The block of this pathway may be due to the increased levels of Bcl-2 able to protect mitochondria functionality. Bcl-2 is considered a potent multidrug resistance factor [40].

Although ROS can play a role in oxaliplatin-induced apoptosis of human renal cancer cells [41], O_2_^.−^ production is not modified by oxaliplatin in HT-29. These human adenocarcinoma cell line shows a basal O_2_^.−^ production higher than astrocytes, suggesting that these tumoral cells are less susceptible to the oxidative toxic effect and can therefore survive in the presence of high ROS concentrations.

The faster catabolism of cancer cells is lesser sustained by mitochondria in comparison to normal non-neoplastic cells [42]. Tumor cells may produce energy by a high rate of glycolysis followed by lactic acid fermentation in the cytosol. On the contrary, normal cells show a comparatively low rate of glycolysis followed by oxidation of pyruvate in mitochondria. Since glycolysis provides most of the building blocks required for cell proliferation, cancer cells need to activate glycolysis [43]. A lesser sensitivity of the mitochondria functionality against oxaliplatin toxicity in HT-29 in comparison to astrocytes is suggested.

## 4. Experimental Section

### 4.1. Cell Cultures

The human colon cancer cell lines HT-29 and the rat adrenal pheochromocytoma cell line PC12 were obtained from American Type Culture Collection (Rockville, MD, USA). HT-29 were cultured in DMEM high glucose (Invitrogen, Milan, Italy, 11965-092) and PC12 in RMPI 1640 (Invitrogen, 11875-085). Medium for HT-29 was supplemented with 10% FBS, 2 mM l-glutamine, 100 IU·mL^−1^ penicillin and 100 μg·mL^−1^ streptomycin (Sigma-Aldrich, Milan, Italy, S6501). Medium for PC12 cells was supplemented with 5% FBS, 10% horse serum, 100 IU·mL^−1^ penicillin and 100 μg·mL^−1^ streptomycin (Sigma-Aldrich, S6501). Cells were maintained at 37 °C and 5% CO_2_ atmosphere.

Primary cultures of astrocytes were obtained according to the method described by McCarthy and de Vellis [44]. Briefly, the cerebral cortex of newborn (P1–P3) Sprague-Dawley rats (Harlan, Padova, Italy) were dissociated in Hanks’ balanced salt solution containing 0.5% trypsin/EDTA and 1% DNase (Sigma-Aldrich, T4174 and D4527) for 30 min at 37 °C. The suspension was mechanically homogenized and filtered. Cells were plated in high-glucose DMEM with 10% FBS. Confluent primary glial cultures were used to isolate astrocytes, removing microglia and oligodendrocytes by shaking. Immunocytochemistry analysis with GFAP staining revealed the purity of astrocyte cultures. Cells were fixed in 4% paraformaldehyde, then incubated with the antibody (Dako, Glostrup, Denmark, Z033429, 1:500), and visualized using Alexa Fluor-conjugated secondary antibody (Invitrogen, Milan, Italy, #A11034, 1:500). Nuclei were stained with 4,6-diamidino-2-phenylindole dihydrochloride. GFAP-positive cells were 90% in astrocyte cultures. Astrocytes were plated, according to the experimental needs, 21 days after cell isolation. Formal approval to conduct the experiments described was obtained from the Animal Subjects Review Board of the University of Florence. The ethics policy of the University of Florence complies with the Guide for the Care and Use of Laboratory Animals of the U.S. National Institutes of Health (NIH Publication No. 85-23, revised 1996; University of Florence Assurance No. A5278-01).

HT-29, PC12 and astrocyte cells were starved in serum-free DMEM overnight before all treatments. Protein concentrations were measured by bicinchoninc acid assay (Sigma-Aldrich, for Bicinchoninic Acid Solution B9643, for Copper sulphate solution C2284).

Oxaliplatin (Sequoia Research Products, Pangbourne, UK), concentrations and times of incubation were chosen on the basis of previously performed evaluations [9].

### 4.2. Cytochrome C Cytosolic Release

Astrocytes or HT-29 cells were plated in d-polylisinated slides (10^5^ cells/slide) and 48 h after were incubated with 100 μM oxaliplatin for 8 h. After treatment, the cells were fixed with 4% paraformaldehyde in PBS, permeabilized for 10 min with PBS containing 0.1% Triton X-100, blocked with PBS containing albumin 1% (Sigma-Aldrich, A2153) and incubated overnight with a rabbit anti-cytochrome C antibody at 4 °C (Santa Cruz Biotechnology, Dallas, TX, USA, sc-7159, 1:300). Slides were washed three times with PBS and incubated with the appropriate secondary antibody labeled with Alexa Fluor 488 (Invitrogen, #A11034, 1:500) at room temperature for 1 h. Images were acquired by a motorized Leica DM6000B microscope equipped with a DFC350FX camera (Leica, Lawrenceville, GA, USA). Cytochrome C cytosolic levels were assessed by inspection of at least three fields (40X 0.75NA objective) *per* slide; two slides for each condition were analyzed. Cells displaying a diffuse cytosolic staining pattern were counted using the “cell counter” plugin of ImageJ (1.33 free-share image analysis software, ImageJ, NIH, Bethesda, MD, USA). Results were expressed as percentage of cells displaying a diffuse (released) distribution of cytochrome C [45,46].

### 4.3. Superoxide Dismutase (SOD)-Inhibitable Superoxide Anion (O_2_^.−^) Levels

Astrocytes and HT-29 cells were plated in six-well plates (5 × 10^5^ cells/well for astrocytes and 3 × 10^5^ cells/well for HT-29) and, 48 h after, they were incubated with or without 100 μM oxaliplatin in serum-free DMEM containing cytochrome C from bovine heart (Sigma-Aldrich, C2037, 1 mg/mL) for 4 h at 37 °C. Non-specific cytochrome C reduction was evaluated by carrying out tests in the presence of bovine SOD (Sigma-Aldrich, S9697, 300 mU/mL). The supernatants were collected, and the optical density was spectrophotometrically measured at 550 nm. After subtracting the non-specific absorbance, the SOD-inhibitable O_2_^.−^ amount was calculated by using an extinction coefficient of 2.1 × 10^4^ M^−1^·cm^−1^ and expressed as µM/mg proteins/4 h. The 4 h incubation interval was chosen on the basis of preliminary experiments, which showed poor reliability for longer cytochrome C exposure to the cellular environment.

### 4.4. Western Blot Analysis

Astrocytes or HT-29 cells were plated in 25 cm^2^ cell culture flasks (10^6^ cells/flask), grown for 48 h and incubated with 100 μM oxaliplatin for 8 h. After treatment, the cells were homogenized in lysis buffer containing 50 mM Tris-HCl pH 8.0, 150 mM NaCl, 1 mM EDTA, 0.5% Triton X-100, Complete Protease Inhibitor (Roche, Milan, Italy, 04693124001), and the homogenate was incubated on ice for 30 min. The suspension was sonicated on ice for 15 s. After centrifugation (13,000× *g* for 15 min at 4 °C), aliquots containing 35 μg total proteins were separated on a 4%–12% sodium dodecyl sulfate (SDS)-polyacrylamide gel by electrophoresis and transferred onto nitrocellulose membranes. Membranes were blocked with 5% nonfat dry milk in PBS containing 0.1% Tween 20 (PBST) and then probed overnight with specific primary antibodies. Goat anti-DR5 receptor antibody (Santa Cruz Biotechnology, sc-19529), rabbit anti Bcl-2 antibody (Cell Signaling, Milan, Italy, #2876) and rabbit anti Bid antibody (Santa Cruz Biotechnology, sc-11423) were diluted to 1:500, 1:1000 and 1:1000, respectively, in 5% nonfat dry milk in PBST. Membranes were washed with PBST and incubated for 1 h in PBST containing the appropriate horseradish peroxidase-conjugated secondary antibody (anti rabbit Cell Signaling, #7074, 1:5000; anti goat Santa Cruz Biotechnology, sc-2020, 1:5000). ECL (Enhanced chemiluminescence; Pierce, Milan, Italy, 32106) was used to visualize the peroxidase-coated bands. Densitometric analysis was performed using the ‘‘ImageJ” analysis software (ImageJ, NIH) and results were normalized to GAPDH (Cell signaling, #2118, 1:2000) immunoreactivity as internal control. Values were reported as percentages in comparison to control, which was arbitrarily fixed at 100%.

### 4.5. Caspase-8 Activity

Astrocytes, HT-29 and PC12 cells were plated in six-well plates (5 × 10^5^ cells/well for astrocytes and 3 × 10^5^ cells/well for HT-29 and PC12) and, after 48 h, they were incubated with 100 μM oxaliplatin for 8 h. After treatment, cells were scraped with 70 µL of lysis buffer as suggested by the manufacturer (Molecular Probes, Milan, Italy). Fifty microliters of the supernatant were incubated with 50 µM of the specific fluorogenic peptide caspase-8 substrate (IETD-AFC composed by 7-amino-4-trifluoromethyl coumarin and a synthetic tetrapeptide Ile-Glu-Thr-Asp) at 25 °C for 30 min. The amount of cleaved substrate in each sample was measured in a 96 well plate fluorescence spectrometer (Flexi Station III, Molecular Devices, Sunnyvale, CA, USA; excitation at 400 nm and emission at 505 nm).

### 4.6. Statistical Analysis

Results were expressed as mean ± SEM and analysis of variance (ANOVA) was performed. A Bonferroni’s significant difference procedure was used as *post hoc* comparison. All assessments were made by researchers blinded to cell treatments. Data were analyzed using the “Origin 8.1” software (OriginLab, Northampton, MA, USA).

## 5. Conclusions 

Taken together the present data underline that oxaliplatin-dependent apoptosis is mediated preferentially by the intrinsic apoptosis pathway in nervous normal cells. The extrinsic pathway based on caspase-8 activation strongly participates to apoptosis phenomena in tumor cells. New neuroprotective agents able to preserve mitochondrial functionality without interfere with caspase-8 activation are suggested as safe candidate to treat oxaliplatin-dependent neurotoxicity.

## Supplementary Materials

Supplementary materials can be found at http://www.mdpi.com/1422-0067/16/03/5386/s1.
