# Supplementary material for: Different Apoptotic Pathways Activated by Oxaliplatin in Primary Astrocytes vs. Colo-Rectal Cancer Cells
Source: Int J Mol Sci. 2015 Mar 9;16(3):5386–99. doi: 10.3390/ijms16035386 (PMC4394482; doi:10.3390/ijms16035386)
Supplement: Supplementary file 1 [file ijms-16-05386-s001.pdf]

Supplementary Information

Table S1. Comparison between astrocyte and HT-29 cell viability after 24 h incubation.

Cell Viability %		
**Oxaliplatin Concentration (μM)**	**Astrocytes**	**HT-29**
0	100.0 ± 0.0	100.0 ± 2.6
0.3	95.4 ± 4.1	97.2 ± 3.5
1	91.0 ± 2.6	92.4 ± 4.6
3	82.3 ± 6.8	90.4 ± 4.2
10	78.6 ± 2.4	85.3 ± 3.8
30	74.6 ± 3.8	79.0 ± 2.9
100	70.2 ± 2.2	65.4 ± 3.5

Astrocyte cells (1×10^4 cells/well) or HT-29 cells (5×10^3 cells) were treated with increasing concentrations of oxaliplatin (1–100 μM). Incubation was allowed for 24 h. Cell viability was measured by MTT assay. Control condition was arbitrarily set as 100% and values are expressed as the mean ± S.E.M. of three experiments.
